# Treatment Patterns, Healthcare Utilization, and Related Costs for Prurigo Nodularis in Sweden

**DOI:** 10.2340/actadv.v105.43730

**Published:** 2025-08-18

**Authors:** Laura VON KOBYLETZKI, Alexandra METSINI, Simon E. REGNELL, Michael CARLBERG, Åke SVENSSON, Annarita ANTELMI

**Affiliations:** 1 Clinical Epidemiology and Biostatistics, School of Medical Sciences, Faculty of Medicine and Health, Örebro University, Örebro; 2 Department of Occupational and Environmental Dermatology, Skåne University Hospital, Lund University, Malmö; 3 Örebro University, School of Medical Sciences, Örebro; 4 Department of Healthcare, Knowledge Support Unit, Region Värmland; 5 Sanofi AB, Stockholm; 6 Department of Dermatology, Lund University, Skåne University Hospital, Malmö, Sweden

**Keywords:** prurigo nodularis, epidemiology, costs, treatment patterns, healthcare resource utilization

## Abstract

Prurigo nodularis (PN) is a chronic, itchy, inflammatory skin condition that negatively affects quality of life. A study was undertaken to investigate the healthcare utilization, including treatment patterns and direct costs for specialist care, for PN in Sweden. Linkage cohorts were created from national Swedish patient and prescription registers, and the cost-per-patient database of PN adults in specialist care in Sweden from 2015 to 2020. Around 875 patients were registered annually with a specialist diagnosis of PN in Sweden, with 3,548 specialist visits per year on average. In patients with severe PN with AD, the most common treatment sequence was topical treatment with corticosteroids followed by systemic prednisolone and methotrexate (32.6%). More than one-fifth of individuals with PN, and most with severe PN, had treatment for more than 1 year. For in- and outpatient care, the mean cost per visit was €458.6 and per patient per year around €1,862. The total annual cost of PN patients is estimated to be €1.6 million in Sweden. A high proportion of patients are treated for years with several, often systemic, treatment sequels. Targeted treatments for PN might improve patients’ quality of life and reduce the high related costs for the healthcare system.

## SIGNIFICANCE

The findings of this study underscore the significant burden of prurigo nodularis on both patients and the healthcare system in Sweden, the high prevalence, and healthcare utilization, with many patients remaining in specialist care for several years despite sequels of systemic treatments. The outcomes suggest that there is a need for targeted effective treatments that could improve patient outcomes, reduce the burden on healthcare system and ensure an efficient resource allocation.

Prurigo nodularis (PN) is a chronic skin condition characterized by pruritic and often hyperkeratotic nodules (1). Scratching has been described as a trigger factor for PN (2). The pathogenesis of PN involves an immune and neural dysregulation resulting in the establishment of a vicious “itch–scratch cycle” that leads to the inflammatory skin lesions (1, 3, 4). PN has been associated with a variety of medical conditions like atopic dermatitis (AD), HIV infection, chronic kidney disease, cardiometabolic disease, neurological diseases, anxiety, and depression (5, 6). PN negatively affects the quality of life of patients in terms of general health, mental health, and economic burden (7).

We recently estimated the cumulative prevalence of adult PN patients in Sweden between the years 2015–2020 to be 49.2 per 100,000 (*n* = 3,253) using the distinct code (L28.1) for PN assigned in 2015 in the International Classification of Diseases, 10th Revision (ICD-10) (8). PN represents a therapeutic challenge. Recent international treatment guidelines suggest a combination of topical and systemic treatments (7, 9–11). Few studies have assessed treatment patterns and healthcare resource utilization for PN and no study assessed those outcomes by severity.

This study aims to assess treatment patterns, outpatient and inpatient specialist healthcare resource utilization, and related costs of PN in patients with and without AD, as well in patients with severe PN, to provide a comprehensive view of the impact of PN on the healthcare system in Sweden.

## MATERIALS AND METHODS

Linkage cohorts were created from large-scale linked data from Swedish patient and prescription registers and the cost-per-patient database of PN adults in specialist care who lived in Sweden in 2015–2020. The registers included more than 10 million individuals, as previously described (8). Data on the diagnosis of PN, the number and type of contacts with healthcare professionals, pharmacological treatments, use of UV therapy (UVA, UVB, PUVA) and balneotherapy, wound dressings and treatments for skin infections, and healthcare costs were analysed. The combinations and sequence of treatments for severe PN were also described. All analyses were performed by age group, sex, and diagnosis of AD, if appropriate.

### Registers

All registers used in this study have been described previously (8). In short, several national registers have been linked by the Swedish National Board of Health and the National Patient Register contains prospectively collected inpatient and outpatient data, including geographical, administrative, and medical data including ICD-10 codes for PN and AD. For all included persons, data on PN were collected (ICD-10: L28.1) from 2015 to 2020.

The Prescription Register compiles prescriptions in primary and secondary care, excluding treatments given in hospital, classified by Anatomical Therapeutic Chemical (ATC) code.

The Total Population Register was used to obtain information regarding participants’ sex and exit due to death or emigration.

The cost-per-patient (CPP) database includes all regions in Sweden and contains information related to healthcare visits, and related diagnoses, types of contact, healthcare measure codes, dates, age, sex, and direct healthcare costs. Costs include treatments (medication, physical treatment) related to specialist healthcare visits and primary care. The database contains over 94% of all care contacts in Swedish specialized inpatient care and about 83% of total specialized outpatient care (12). For this study, data from the CPP database (inpatient and outpatient care) were extracted for the period 2015–2022, as a distinct ICD code for PN was introduced in 2015.

### Severe prurigo nodularis

Severe PN was defined, according to expert opinion (8), as the use of 1 or a combination of systemic treatments given at the same time as or after PN diagnosis.

### UV therapy, balneotherapy, and wound dressing

The Swedish Classification of Healthcare Measures is a mandatory coding system for interventions by healthcare professionals, including medical, surgical, and diagnostic areas (13).

### Statistical analysis

Cross-tabulation and means were used to describe the study population, including PN prevalence and medication use. Disease prevalence was described for PN overall and for severe PN and stratified by year and by diagnosis of AD. Stratified analyses were also performed by age group and sex, if relevant. Data for 2020 were compared with previous years to indicate whether the COVID-19 pandemic affected resource consumption.

A χ^2^ test was used to assess differences in prevalences between groups. For example, the test evaluated whether there was a difference in cumulative prevalence of PN across age groups. A *p*-value below 0.05 indicated statistical significance.

The CPP database was analysed to define the number of patients accessing the variety of healthcare services for the diagnosis, the treatment, and the follow-up of PN and severe PN. Specifically, the number of visits and their frequency, the costs per visit and treatment, the treatment patterns, the patient’s follow-up year after year and all the relative costs. The type of contacts (e.g., physical visit, telephone contact), the type of visits with the healthcare personnel (e.g., dermatologist, general practitioner, psychologist, nurse, etc.) and treatments measures (e.g., lubrication, UV therapy, medical baths) were categorized and assessed in term of frequency and costs. All analyses were performed by sex and age group.

Healthcare costs were calculated per visit, by profession, by type of contact, per patient per year, for outpatient care only, as well as inpatient and outpatient care together.

Standard deviations and min/max values were reported. The costs were calculated, the cost values were adapted to 2022 price level based on the Swedish consumer price index (mean annual inflation rate between 2015 and 2022 was 1.32%) converted to prices in Euro using the average annual exchange rate in 2021 (1 SEK = €0.0985 EUR). The analysis was conducted using Microsoft Excel (Microsoft Corp, Redmond, WA, USA) and Stata/MP 17.0 (StataCorp LLC, College Station, TX, USA) statistical software.

### Ethical approval

The research project (2021-04149) was approved by the Swedish Ethical Review Authority in Uppsala (approval number dnr 2019-04807, 2021-04149, 2022-04573-02).

## RESULTS

In total, 3,253 patients aged 18 years and older had a diagnosis of PN and 6,607,787 had no diagnosis of PN during the study period.

### Prevalence of prurigo nodularis

Of all adult persons with a diagnosis of PN in specialist care during 2015 to 2020 (*n* = 3,253), 46.4% (n = 1,510) had severe PN, with a cumulative prevalence of 22.8 per 100,000, and with the age group 80+ being overrepresented. (Table I). Nearly half of individuals with severe PN had AD, and 50.9% had no comorbid AD; the difference was not statistically significant (χ^2^ test, *p* = 0.06). There was no statistically significant difference in the PN prevalence between pre-pandemic years (2015–2019) and 2020 (*p* > 0.05).

**Table I:** Cumulative prevalence of severe prurigo nodularis (PN), 2015-2020, ≥ 18 years old

Item	No. severe PN^a^	No. total	Severe PN per 100,000	Percentage of all PN	*p*-value^*^
All	1,510	6,611,040	22.8	46.4	
Sex					0.31
Male	638	2,920,935	21.8	45.1	
Female	872	3,690,105	23.6	47.4	
Comorbid AD					< 0.001
Non-AD	305	5,354,972	5.7	47.9	
AD	1,205	1,256,068	95.9	46.1	
Age groups					< 0.001
18–39	93	2,117,681	4.4	41.9	
40–59	328	1,973,528	16.6	42.2	
60–79	767	1,987,286	38.6	47.0	
80+	322	532,545	60.5	51.7	

a Defined as having treatment with any of the following systemic treatments or any combination of the treatments: oral corticosteroids, cyclosporine, or gabapentinoids using the ATC codes L04AD01, N03AX12, N03AX16, H02AB01, H02AB06, H02AB07, H02AB08, D11AH05, L04AX03, L04AX02, L04AX04, L04AA06. Individuals with and without topical treatment are included. Individuals with last treatment before first PN diagnosis are not included as severe PN.

* χ^2^ test, prevalence.

The cumulative prevalence (2015–2020) of adult severe PN patients is displayed in Table II.

**Table II:** Cumulative prevalence of severe prurigo nodularis (PN) for patients treated with topical corticosteroids, groups II–IV 2015–2020, ≥ 18 years old

Item	No. severe PN^a^	No. total	Severe PN per 100,000	Percentage of all with PN+topical corticosteroids	*p*-value^*^
All	1,481	6,611,040	22.4	47.2	
Sex					0.17
Male	628	2,920,935	21.5	46.0	
Female	853	3,690,105	23.1	48.2	
Comorbid AD					< 0.001
Non-AD	287	5,354,972	5.4	50.9	
AD	1,194	1,256,068	95.1	46.4	
Age groups					< 0.001
18–39	90	2,117,681	4.2	43.7	
40–59	323	1,973,528	16.4	43.6	
60–79	752	1,987,286	37.8	47.7	
80+	316	532,545	59.3	51.7	

a Defined as having treatment with any of the following systemic treatments or any combination of the treatments: oral corticosteroids, cyclosporine, or gabapentinoids using the ATC codes L04AD01, N03AX12, N03AX16, H02AB01, H02AB06, H02AB07, H02AB08, D11AH05, L04AX03, L04AX02, L04AX04, L04AA06. Only individuals with groups II–IV topical treatment with corticosteroids are included. Individuals with last treatment before first PN diagnosis are not included as severe PN. For individuals with severe PN in 2015, topical treatment was also included if that occurred in the year 2014.

* χ^2^ test, prevalence.

### Treatment of prurigo nodularis

The cumulative prevalence (2015–2020) of adult severe PN patients using topical corticosteroids group II–IV and systemic treatment is presented in Table II. The most common systemic treatments in severe PN patients (*n* =1,510) were systemic glucocorticoids and methotrexate, used by 78.7% and 38.7% of patients respectively; 47.2% of all PN patients treated with topical corticosteroids had severe PN (Table II).

Some 50.9% of patients without AD and 46.4% of those with AD had severe PN; the difference was not statistically significant (*p*, χ^2^ test = 0.06). In total, there were more than 100 treatment combinations in the study group; 1,481 patients with severe PN treated with topical corticosteroids in groups II–IV were simultaneously treated with systemic prednisolone in monotherapy (20.5%) or with the combination prednisolone and methotrexate (12.1%). These combined treatments were the most common in severe PN patients with a diagnosis of AD (*n* = 1,194; Table III).

**Table III:** Most common combinations of treatments (all, ***n*** ≥ 10) included in severe prurigo nodularis (PN)^a^, 2015–2020, ≥ 18 years old, for patients treated with topical corticosteroids, groups II–IV

Item	All (*n* =1,481)		No AD (*n* =287)		AD (*n* =1,194)	
	*n*	%^b^	*n*	%^b^	*n*	%^b^
Prednisolone only	304	20.5	37	12.9	267	22.4
Prednisolone + methotrexate	179	12.1	41	14.3	138	11.6
Betamethasone + prednisolone	132	8.9	21	7.3	111	9.3
Methotrexate only	125	8.4	36	12.5	89	7.5
Betamethasone only	90	6.1	16	5.6	74	6.2
Gabapentin only	57	3.8	14	4.9	43	3.6
Betamethasone + prednisolone + methotrexate	53	3.6	10	3.5	43	3.6
Gabapentin + prednisolone	48	3.2	8	2.8	40	3.4
Pregabalin only	33	2.2	5	1.7	28	2.3
Betamethasone + methotrexate	27	1.8	6	2.1	21	1.8
Gabapentin + betamethasone +prednisolone	26	1.8	3	1.0	23	1.9
Gabapentin + prednisolone +methotrexate	24	1.6	8	2.8	16	1.3
Pregabalin + prednisolone	19	1.3	2	0.7	17	1.4
Gabapentin + methotrexate	19	1.3	7	2.4	12	1.0
Cyclosporine + prednisolone +methotrexate	17	1.1	3	1.0	14	1.2
Cyclosporine + prednisolone	17	1.1	3	1.0	14	1.2
Prednisolone + mycophenolic acid	16	1.1	1	0.3	15	1.3
Gabapentin + betamethasone	15	1.0	3	1.0	12	1.0
Pregabalin + prednisolone +methotrexate	13	0.9	4	1.4	9	0.8
Cyclosporine + methotrexate	13	0.9	4	1.4	9	0.8
Cyclosporine only	12	0.8	2	0.7	10	0.8
Pregabalin + betamethasone	11	0.7	0	0.0	11	0.9

a Defined as having treatment with any of the following systemic treatments or any combination of the treatments: oral corticosteroids, cyclosporine, or gabapentinoids using the ATC codes L04AD01, N03AX12, N03AX16, H02AB01, H02AB06, H02AB07, H02AB08, D11AH05, L04AX03, L04AX02, L04AX04, L04AA06. Only individuals with groups II–IV topical treatment with corticosteroids are included. Individuals with last treatment before first PN diagnosis are not included as severe PN. For individuals with severe PN in 2015, topical treatment was also included if that occurred in the year 2014.

b Percentage of severe PN+D07AB-D07AD.

**Fig. 1:**
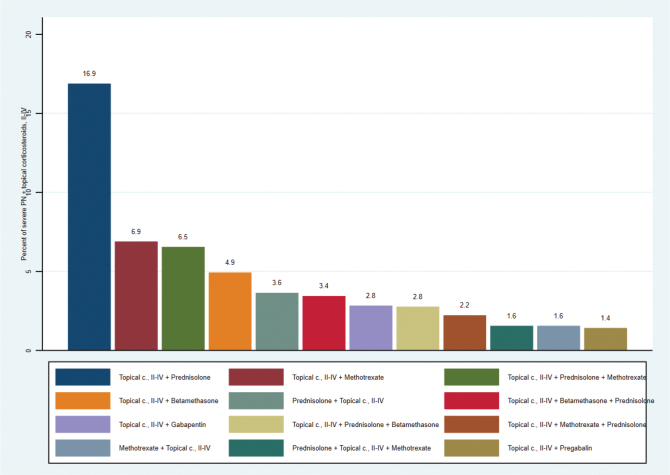
Most common treatment pathways among patients with severe prurigo nodularis (PN)^a^, 2015–2020, ≥ 18 years old (***n*** =1,481). The systemic treatments are displayed in sequence and combined with topical corticosteroids. Only individuals with groups II–IV topical treatment with corticosteroids are included***.***^a^Defined as having treatment with any of the following systemic treatments or any combination of the treatments: oral corticosteroids, cyclosporine, or gabapentinoids.

When a systemic treatment was used as second line after insufficient effect of topical treatment with corticosteroids in groups II–IV, prednisolone was the most prescribed drug (16.9%), followed by methotrexate (6.9%) (Fig. 1). The most common number of sequential treatments was as follows: 488 (32.9%) patients had 1, 274 (18.5%) had 2, and 23 (1.5%) had 3 additional treatments (Fig. 1, Table SI).

The treatment modalities were not supported by Swedish national guidelines but were instead based on clinical practice, and therefore not fully aligned with the most recent international guidelines for the treatment of PN.

### Data from cost-per-patient

About 7,000 patients were registered in the CPP dataset with a diagnosis of PN in the period 2015–2022 (Tables SII and SIII). Annually, about 875 patients were registered with a diagnosis of PN. The total number of (unique) patients with a diagnosis of PN in specialist (inpatient and outpatient) care was 4,180. The number of unique patients in outpatient care only was 4,088. The vast majority of PN patient visits were outpatient visits.

The proportion of females and males was equally distributed (*p* = 0.12), and females were slightly younger (mean age 64 vs 65, *p* < 0.05). The same held for inpatient care (51% males vs 49% females, *p* = 0.57) with similar age distribution (females 69.4 and males 70.5 years old, *p* = 0.52). In outpatient care, there was an equal distribution of females and males (53% females vs 47% males, *p* = 0.95), and the mean age was 65 years (females 64.8 and males 65.9 years old, *p* < 0.05).

Individuals with PN had 4.1 healthcare visits per year on average. In total, these patients had over 28,000 healthcare visits per year, mainly in specialist outpatient care (see Table SII). The vast majority (95%) of visits took place in the dermatology clinic (see Table SIII). Physicians (42%) and nurses (40%) are the main healthcare professionals that the patients visit, followed by assistant nurses (17%) (Table SIV). Some 31.7% of patients in outpatient care received some sort of phototherapy and 7.3% lubrication therapy (Table SV).

### Outpatient care

In outpatient care, the cost per type of contact was higher for team visits (€555.8), followed by single visits (€249.5) and telephone contact (€216.3). However, the number of physician visits was higher than team visits (Table SVI). The average cost per visit was €247.5.

The most expensive type of caregiver visit was with physicians (€348.7), followed by psychotherapists (€259.7). Visits to nurses, psychologists, assistant nurses, and other categories of caregivers cost a similar amount, except for dietitians, who had a lower cost per visit (€58.6). The average cost per visit was €247.5 (Table SVII).

### Inpatient care

An average of almost 72 inpatient visits involving 56 patients with a main diagnosis of PN took place per year in Sweden. The average number of yearly visits for these patients was 1.3. The average cost per visit was almost €10,631, corresponding to €13,755 per patient per year, thus higher than outpatient care (*p* < 0.05 in both cases) (Table SVIII).

### Inpatient and outpatient care

A total of 3,548 inpatient and outpatient visits took place per year on average, involving 875 patients per year; 4.1 visits per patient took place on average per year. The average cost per visit was €458.6 and per patient per year around €1,862 (Table SIX). The total annual cost of PN patients is estimated at around €1.6 million. Of all patients with PN in 2015, 67% received treatment in specialist care during 2015 only, whereas all other patients received specialist care for 2 or more years (Table SX).

## DISCUSSION

This longitudinal nationwide linkage study is the first in Sweden to report the burden of PN, especially when severe, including treatment patterns, healthcare utilization, and related costs in a general population of adults in specialist healthcare.

The cumulative 5-year prevalence of severe PN in adults was high, at 22.8 per 100,000. A large number of these patients underwent different systemic treatments and healthcare specialist visits for several years. The yearly prevalence of PN ranged between 10.6 and 12.4 per 100,000 patients. Adult PN patients accounted for over 24,000 healthcare visits between 2015 and 2020 in Sweden. The number of healthcare contacts per year was quite high, considering that 875 patients registered with a diagnosis of PN collectively had 3,548 visits per year on average, i.e., 4.1 visits per year per patient. Physicians were the type of healthcare professional who most frequently encountered PN patients (10,162 visits), followed by nurses (9,832 visits). AD, female sex, and older age predicted severe PN and changes in treatment, entailing several systemic treatments and specialist healthcare visits over several years.

The most preferred systemic treatment was prednisolone, followed by methotrexate. This was likely due to the absence of Swedish national guidelines, leading the treatment choice to rely largely on individual clinical experience and established practice. The choice of prednisolone was driven by the need to provide fast, albeit short-term, symptom relief for patients. However, this approach has likely underestimated the risks of entrapping patients in a cycle of flare-ups and regressions, which in turn may lead to increased healthcare visits and a greater demand for medical resources.

The treatment of PN notably resulted in quite high costs. However, despite intensive treatment, a high proportion of individuals with PN remained in specialist care for more than 1 year and even, in some instances, over 5 years. Almost half of patients received systemic treatments. About one-third of patients underwent treatment changes and a sequence of several systemic treatments or episodic management with oral steroids, probably due to not reaching treatment goals.

The results of the prevalence of PN from the current study are slightly higher than indicated by other studies performed in Europe (1, 9, 14–15), Previous studies have estimated a wide range of prevalence due to marked differences in study populations included, varying case definitions, ethnic differences, and different time frames considered (16). For instance, a study from the UK demonstrated a prevalence of 3.27 per 100,000 patients, using representative data from the National Health Service. The estimated 1-year (2018) prevalence (L28.1) of inpatients at an academic dermatology clinic in Poland was relatively high, at 6.52 cases per 100,000, as the study was based on more selected tertiary care patients (9, 14).

In a study by Ständer et al. on the prevalence of PN in the general US population, the 1-year prevalence was estimated at 36.7–43.9 per 100,000 from October 2017 to September 2018 (17). Misery et al. estimated the prevalence of PN (L28.1) at 8.4 per 100,000 population and of other prurigo (L28.2) at 46.7 per 100,000 among in- and outpatients at a French itch expert centre (18).

Few studies have examined healthcare utilization in PN. In England a high burden of healthcare utilization was estimated, especially in primary and in secondary care, regarding in particular moderate-to-severe PN (19). Wang et al. found that stays in the hospital for PN patients in the USA in 2016 were longer and costlier than for patients without PN (20). Huang et al. described the real-world healthcare utilization of PN patients in the USA over 15 months between 2015 and 2016 (21), comparing PN with matched control groups with psoriasis and AD. Patients with PN were more than 30 times as likely to be seen by a dermatologist compared with controls and more than 5 times as likely as those with AD or psoriasis; however, they did not observe statistically significant differences in healthcare costs between PN patients and control groups. Conversely, another study in the United States showed that PN was associated with higher utilization of specialty care and a comorbidity burden than age- and sex-matched controls, as well as compared with patients with AD and psoriasis (22).

A recent retrospective study by Ständer et al. was based on data from the German Statutory Health Insurance (which represents nearly 90% of all German inhabitants). Healthcare costs for patients with an initial PN diagnosis between 2012 and 2016 were compared with those of a reference population containing a cross-section of healthy and unhealthy individuals in Germany. The authors reported significantly higher average initial costs for PN inpatients (€760.38 vs €320.55) and outpatients (€330.15 vs €149.54), as well as drug costs (€352.50 vs €179.94) compared with patients without PN (23).

### Strengths and limitations

The strengths of this study include the use of nationwide, prospectively recorded register data, a longitudinal design, and a comparison design confirming the frequency of PN cases. By using 2 cohorts, we derived similar estimates of the prevalence of adults with a diagnosis of PN from different data sources.

However, the study has potential limitations. The current results might still underestimate the prevalence of PN. Not all physicians use ICD-10 for the diagnosis of PN, especially when there are comorbidities. For example, in PN patients with AD, often only AD is registered as a diagnosis. Furthermore, some physicians might use other diagnoses instead of the designated ICD-10 code to define a clinical picture of PN, particularly in primary care where many patients with mild or moderate PN can be managed for a long time before being referred to a specialist.

Additionally, not all PN patients were reported in the CPP database, mainly due to missing and incomplete primary care data. Primary care data have been added to the CPP database since 2019, but not all regions have been reporting these data, and the diagnosis of PN is often missed in primary care (20). The study assessed the prevalence of PN based on specialist diagnosis, increasing the reliability of the results. PN diagnosis is difficult and requires specialist assessment; in particular, mild cases may be missed in primary care and not be referred to a specialist. Thus, the total prevalence of PN may be higher. Furthermore, it cannot be ruled out that some costs to some extent, due to the structure of the database and overlapping diagnoses, could reflect treatment of comorbidities, rather than PN alone.

### Generalizability

The study’s findings, although specific to Sweden, have broader implications for similar healthcare systems. Despite the differences in the prevalence estimates between populations, the slightly higher prevalence rates align with US data and data from some European countries, suggesting a comparable burden of PN across populations. Consistent patterns of healthcare utilization and costs indicate a universal need for better PN management strategies.

Key predictors for severe PN, such as AD, female sex, and older age, are likely applicable elsewhere, enabling the identification of high-risk patients and tailoring treatment strategies. The economic burden highlighted is important for healthcare policymakers, informing cost-effectiveness and budget allocation. By considering these aspects, healthcare providers and policymakers can better manage PN and improve patient outcomes on a broader scale.

### Conclusions

PN, especially when severe, accounts for high healthcare utilization including costs and low quality of life. Most individuals with severe PN are treated for more than 1 year with several different treatments. Risk groups, such as those with severe PN, may need more effective management strategies. Targeted treatments for PN might improve clinical outcomes and patient safety profiles, decreasing the overall disease burden for patients and high related costs for the healthcare system in the long term.

## Supplementary Material


