# Supplementary material for: Treatment Patterns, Healthcare Utilization, and Related Costs for Prurigo Nodularis in Sweden
Source: Acta Derm Venereol. 2025 Aug 18;105:43730. doi: 10.2340/actadv.v105.43730 (PMC12372295; doi:10.2340/actadv.v105.43730)
Supplement: Supplementary file 1 [file ActaDV-105-43730-s1.pdf]

Supplementary material has been published as submitted. It has not been copyedited, or typeset by Acta Dermato-Venereologica

Table SI. Most common treatment pathways/order of treatment included in severe prurigo nodularis (PN)[a], 2015-2020, ≥18 years old, for those also treated with topical corticosteroids, group II-IV

All (n=1,481)

First treatment	Second treatment	Third treatment	Fourth treatment	n	%[b]	n, 2020	Percent not finished with treatment at the end of the year 2020[c]
Topical corticosteroids, II-IV	Prednisolone			250	16.9	173	69.2
Topical corticosteroids, II-IV	Methotrexate			102	6.9	84	82.4
Topical corticosteroids, II-IV	Prednisolone	Methotrexate		97	6.5	86	88.7
Topical corticosteroids, II-IV	Betamethasone			73	4.9	47	64.4
Prednisolone	Topical corticosteroids, II-IV			54	3.6	46	85.2
Topical corticosteroids, II-IV	Betamethasone	Prednisolone		51	3.4	40	78.4
Topical corticosteroids, II-IV	Gabapentin			42	2.8	36	85.7
Topical corticosteroids, II-IV	Prednisolone	Betamethasone		41	2.8	37	90.2
Topical corticosteroids, II-IV	Methotrexate	Prednisolone		33	2.2	31	93.9
Methotrexate	Topical corticosteroids, II-IV			23	1.6	19	82.6
Prednisolone	Topical corticosteroids, II-IV	Methotrexate		23	1.6	22	95.7
Topical corticosteroids, II-IV	Pregabalin			21	1.4	15	71.4
Prednisolone	Topical corticosteroids, II-IV	Betamethasone		19	1.3	18	94.7
Betamethasone	Topical corticosteroids, II-IV			17	1.1	12	70.6
Gabapentin	Topical corticosteroids, II-IV			15	1.0	13	86.7
Topical corticosteroids, II-IV	Betamethasone	Methotrexate		15	1.0	10	66.7
Betamethasone	Topical corticosteroids, II-IV	Prednisolone		14	0.9	9	64.3
Topical corticosteroids, II-IV	Gabapentin	Prednisolone		14	0.9	12	85.7
Topical corticosteroids, II-IV	Prednisolone	Ciclosporin		13	0.9	10	76.9
Methotrexate	Topical corticosteroids, II-IV	Prednisolone		13	0.9	12	93.3
Topical corticosteroids, II-IV	Betamethasone	Prednisolone	Methotrexate	13	0.9	11	84.6
Pregabalin	Topical corticosteroids, II-IV			12	0.8	10	83.3
Topical corticosteroids, II-IV	Prednisolone	Methotrexate	Betamethasone	10	0.7	8	80.0
Topical corticosteroids, II-IV	Prednisolone	Gabapentin		10	0.7	9	90.0

[a]*Defined as having treatment with any of the following systemic treatments or any combination of the treatments: oral corticosteroids, cyclosporine or gabapentinoids using the ATC codes L04AD01, N03AX12, N03AX16, H02AB01, H02AB06, H02AB07, H02AB08, D11AH05, L04AX03, L04AX02, L04AX04, L04AA06. Only individuals with group II-IV topical treatment with corticosteroids are included. Individuals with last treatment before first PN diagnosis are not included as severe PN. For individuals with severe PN in 2015, topical treatment was also included if that occurred in the year 2014*

[b]*Percent of severe PN + D07AB-D07AD*

[c]*Percent of pathway combination still in treatment at the end of the time (2020)*

Table SII. Number of patients and visits with a diagnosis of prurigo nodularis in Sweden registered in the cost-per-patient (CPP) dataset per year (inpatient and outpatient care)

Year	Number of patients[a]	Number of visits
2015	636	2657
2016	833	3403
2017	920	3886
2018	941	3961
2019	911	3914
2020	854	3306
2021	931	3484
2022	961	3706
Total	7000	28385
**Mean per year**	**875**	**3548**

[a]Patients can occur in different years

Table SIII. Number of patients and visits per specialist clinic registered in the cost-per-patient (CPP) dataset

Specialist Clinic	Number of patients	Number of visits	Percentage of total visits
Admission/Acute	12	12	0.0%
Emergency Clinic	2	2	0.0%
Internal medicine	92	254	1.1%
Gastroenterology	10	10	0.0%
Cardiovascular	4	4	0.0%
Hematology	8	8	0.0%
Lung	13	18	0.1%
Infection	219	602	2.5%
Rheumatology	5	6	0.0%
Allergy	6	6	0.0%
Renal medicine	17	31	0.1%
Endocrinology	3	3	0.0%
Dermatology	7,416	23,111	95.6%
Occupational Dermat.	25	42	0.2%
Neurology	2	2	0.0%
Cardiology	4	4	0.0%
Geriatrics	5	7	0.0%
LAH/SAH	2	2	0.0%
Geriatric rehab.	1	1	0.0%
Surgery	7	7	0.0%
Orthopedics	10	10	0.0%
Hand surgery	1	1	0.0%
Urology	2	2	0.0%
Transplantation	1	1	0.0%
Gynecology	3	3	0.0%
Obstetrics & gynecology	3	3	0.0%
Ophthalmology	1	1	0.0%
Ear, nose & throat	2	3	0.0%
Medical rehab.	1	1	0.0%
Sports medicine	1	2	0.0%
Oncology, general	3	3	0.0%
Curator	6	16	1.1%

Table SIV. Number of visits per patient per health care professionals and type of visit registered in the cost-per-patient (CPP) dataset

Health care professionals - type of contact	Number of patients	Number of visits	Number of visits per patient	As % of outpatient visits
**Dietitian**	**1**	**2**	**2.00**	**0.0**
Visit	1	2	2.00	0.0
**Psychotherapist**	**31**	**56**	**1.81**	**0.2**
Individual visit	27	45	1.67	0.1
Telephone contact	4	11	2.75	0.0
**Physician**	**6,440**	**10,162**	**1.58**	**42.0**
Individual visit	5420	8544	1.58	35.3
Group visit	20	40	2.00	0.1
Telephone contact	952	1,519	1.60	6.2
Team visit	48	59	1.23	0.2
**Psychologist**	**5**	**20**	**4.00**	**0.0**
Individual visit	5	20	4.00	0.0
**Nurse**	**1,011**	**9,823**	**9.72**	**40.6**
Individual visit	979	9,783	9.99	40.4
Telephone contact	32	40	1.25	0.1
**Assistant nurse**	**387**	**4,052**	**10.47**	**16.7**
Individual visit	386	4,051	10.49	16.7
Telephone contact	1	1	1.00	0.0
**Other**	**12**	**63**	**5.3**	**0.2**
Individual visit	12	63	5,3	0.2

Table SV. Number of visits for various treatments registered in the cost-per-patient (CPP) dataset

Various treatments	Number of visits	As % of outpatient visits	Number of patients	As % of patients (outpatient car n=3649)
Phototherapy, PUVA<br>Phototherapy PUVA, bath	943	3.9%	67	1.8
Phototherapy, UVA	143	0.6%	16	0.4
Phototherapy, UVA1	58	0.2%	7	0.2
Phototherapy, UVB	9,718	40.2%	978	26.8
Phototherapy, UVB and UVA	682	2.8%	80	2.2
Other specified skin phototherapy	18	0.1%	2	0.1
Lubrication	4,136	17.1%	268	7.3
**Total**	**15,698**	**64.9%**	**1,418**	**38.9**

Table SVI. Number of visits, type of contact and related costs (Euros)

Contact type	Number of visits	Health care cost per contact type (mean)	Standard deviation cost	Max	Min
Visit	25 735	249.5	469.4	69 283.8	39.0
Group visit	121	154.0	110.7	777.3	49.3
Telephone contact	1 888	216.3	164.6	2 357.3	45.8
Team visit	64	555.8	387.7	2 348.9	123.4
**Total**	**27 808**	**458,6**	**2 128,9**	**89 288,8**	**39,0**

Table SVII. Number of visits, and related costs (Euros) by health care provider

Caregiver	Number of visits per caregiver	Costs per caregiver (mean)
Dietician	2	58.6
Psychotherapist	70	259.8
Physician	11 754	348.7
Psychologist	22	175.8
Nurse	11 092	179.8
Assistant nurse	4 757	156.9
Other	111	183.7
**Total**	**27 808**	**247.5**

Table SVIII. Number of visits, number of patients. costs (Euros) per visit and per patient (only inpatients)

Year	Number of visits	Number of patients	Number of visits per patient	Mean cost per visit	Max	Min	Mean cost per patient	Max	Min
**2 015**	56.0	42.0	1.3	11 248.4	35 731.1	2 217.9	14 997.9	47 641.4	2 957.2
**2 016**	70.0	50.0	1.4	10 457.7	57 939.9	1 587.2	14 640.8	81 115.9	2 222.0
**2 017**	97.0	76.0	1.3	9 269.5	28 659.1	1 413.7	11 830.8	36 578.1	1 804.3
**2 018**	76.0	62.0	1.2	9 338.9	89 288.8	892.2	11 447.6	109 450.7	1 093.7
**2 019**	66.0	51.0	1.3	10 425.3	42 176.1	1 639.0	13 491.6	54 580.9	2 121.1
**2 020**	66.0	52.0	1.3	12 154.1	58 943.5	1 912.2	15 426.4	74 812.9	2 427.0
**2 021**	70.0	52.0	1.3	11 289.9	44 099.4	1 127.3	15 197.9	59 364.6	1 517.5
**2 022**	76.0	63.0	1.2	11 611.9	77 357.6	2 228.9	14 008.0	93 320.3	2 688.9
**Mean per year**	**72.1**	**56.0**	**1.3**	**10 630.6**	**54 181.4**	**1 599.5**	**13 755.1**	**64 062.8**	**1 891.2**

Table SIX. Number of visits, number of patients. Costs (Euros) per visit and per patient (in- & outpatients)

Year	Number of visits	Number of patients	Number of visits per patient	Mean cost per visit	Max	Min	Mean cost per patient	Max	Min
**2015**	2657	637	4.2	419.2	35 731.1	49.3	1 748.4	149 038.3	205.5
**2016**	3474	847	4.1	407.6	57 939.9	52.3	1 671.8	237 642.5	214.4
**2017**	3886	919	4.2	434.1	28 659.1	56.7	1 835.8	121 185.4	239.7
**2018**	3960	941	4.2	407.1	89 288.8	45.8	1 713.2	375 752.9	192.6
**2019**	3914	910	4.3	423.4	42 176.1	49.7	1 821.0	181 403.8	213.6
**2020**	3305	854	3.9	548.6	69 283.8	71.9	2 123.2	268 129.8	278.3
**2021**	3483	931	3.7	528.8	44 099.4	39.0	1 978.2	164 982.0	145.9
**2022**	3706	961	3.9	506.1	77 357.6	55.8	1 951.5	298 321.9	215.1
**Mean per year**	**3 548.1**	**875.0**	**4.1**	**458.6**	**56 209**	**52.4**	**1 861.7**	**227 931.3**	**212.7**

Table SX. Number and proportion of patients by years of follow up

**Years follow up**	**1**	**2**	**3**	**4**	**5**	**6**	**7**	**8**
Number of patients that finished follow up in the year above	2809	711	281	166	86	69	39	18
Percentage (%) that finished follow up in the year above	67.22	17.01	6.72	3.97	2.06	1.65	0.93	0.43
